# Hyperthermic Intrathoracic Chemoperfusion for Malignant Pleural Mesothelioma: Systematic Review and Meta-Analysis

**DOI:** 10.3390/cancers13143637

**Published:** 2021-07-20

**Authors:** Tommi Järvinen, Juuso Paajanen, Ilkka Ilonen, Jari Räsänen

**Affiliations:** 1 Department of General Thoracic and Esophageal Surgery, Heart and Lung Center, Helsinki University Hospital, 00029 Helsinki, Finland; ilkka.ilonen@hus.fi (I.I.); jari.rasanen@hus.fi (J.R.); 2 Department of Surgery, Clinicum, University of Helsinki, 00029 Helsinki, Finland; 3 Department of Pulmonary Medicine, Heart and Lung Center, Helsinki University Hospital and University of Helsinki, 00029 Helsinki, Finland; juuso.paajanen@hus.fi

**Keywords:** malignant pleural mesothelioma, hyperthermic intrathoracic chemoperfusion

## Abstract

**Simple Summary:**

Treatment of malignant mesothelioma with high-temperature chemotherapeutic instillation of the affected pleural space seems to be advantageous, but higher-quality studies are needed.

**Abstract:**

Malignant pleural mesothelioma (MPM) is an aggressive malignancy of the pleural lining with exceptionally poor survival. Hyperthermic intrathoracic chemoperfusion (HITHOC) is commonly used with surgery in limited disease. However, data on its effect on survival are limited. In this systematic review and meta-analysis, we analyzed a total of 11 observational articles. HITHOC was compared to control arm that did not receive HITHOC in three studies including 762 patients. The pooled analysis of these studies revealed an SMD of 0.24, with 95% CI of 0.06–0.41 favoring the HITHOC group, reaching statistical significance. The survival effect of HITHOC in epithelioid MPM vs. non-epithelioid MPM was analyzed in four studies. Pooled analysis showed an SMD of 0.79 (95% CI = 0.48–1.10) favoring epithelioid MPM. Based on available data, there seems to be a benefit with HITHOC in regards to overall survival in the treatment of all mesothelioma patients. Multicenter randomized controlled trials are needed to validate and standardize this treatment approach.

## 1. Introduction

Malignant pleural mesothelioma (MPM) is an aggressive malignancy of the pleural lining with exceptionally poor survival. Median survival from diagnosis is less than 12 months [1].

Higher than background exposure to asbestos is linked to approximately four fifths of MPM in the Western world; however, the development of MPM in all patients exposed to asbestos is very rare. This leads to a conclusion that additional factors also play a role. A DNA tumor virus, simian virus 40 (SV40), has been implicated in human MPM, as the SV40 large tumor antigen is expressed in mesothelioma cells, but not in nearby stromal cells [2]. Asbestos appears to amplify the transformative effects of SV40 on human mesothelial cells, which supports the hypothesis that SV40 and asbestos are cocarcinogens [3].

The incidence of MPM is rising globally, except in the United States, and the peak incidence is not expected to occur globally for another 5–15 years [4]. Anticipated peaks in Europe and Australia are not predicted to occur for another 5–10 years. In Japan and other non-Western countries, the peak is expected to be delayed compared to Western countries, as widespread use of asbestos in construction occurred later [3].

In cases of limited disease, extensive tumor extraction can be achieved with either extrapleural pneumonectomy (EPP) or extended pleurectomy/decortication (P/D) operations, techniques of which have been previously described [5]. The aim of radical surgery is to achieve macroscopic complete resection (MCR). In addition to surgery, adjuvant therapies, such as chemoradiation, intrapleural chemotherapy, and photodynamic therapy, are used [6,7,8]. Recurrence after only surgical treatment is common and, thus, multimodality treatment including surgery is the mainstay of curative intent treatment, and, in some countries, surgery is not recommended for MPM [9].

Intraoperative intrapleural injection of cytotoxic drugs, such as cisplatin, doxorubicin, gemcitabine, or epirubicin, with hyperthermic perfusion at the time of surgery, i.e., hyperthermic intrathoracic chemotherapy (HITHOC), is a widely used method of multimodality treatment for MPM to optimize local disease control [7,9,10]. A well-described technique of HITHOC is as follows: first, an extended right or left thoracotomy is performed and usually accompanied by a resection of the seventh rib. Then, before the administration of intrapleural chemotherapy, surgical thoracic cytoreduction, usually either P/D or EPP, is performed in an attempt to remove all macroscopic evidence of malignant disease. The pleurectomy involves all parietal and visceral pleural surfaces, including the diaphragm and fissures [11]. This can be accompanied by resection of pericardium and/or diaphragm. In EPP, the ipsilateral lung is removed with the pleural cavity [5]. Some surgeons use diaphragm- and pericardium-sparing partial pleurectomy as the cytoreductive operation of choice [12,13]. Hemostasis and irrigation are then performed and, typically, a partial closure of skin at the thoracotomy site and elevation of skin flaps are performed to form a reservoir. Then, a catheter is inserted through the thoracotomy for infusion of the chemotherapy solution, and a drainage thoracostomy (28 French) tube is inserted at the level of the diaphragm and aimed to the apex of the thoracic cavity. A heated chemotherapeutic agent is circulated by a hyperthermia pump. The ipsilateral lung is maintained at a partially collapsed state during this infusion [11]. Potential benefits of HITHOC include enhanced local cytotoxic effects on tumor cells, with limited systemic side effects [14,15]. Despite its wide usage and its well-established counterpart of hyperthermic intraperitoneal chemotherapy (HIPEC) in the treatment of peritoneal carcinomatosis and pseudomyxoma peritonei, relatively little evidence exists on the benefits of HITHOC in the treatment of MPM [16].

The aim of this systematic review and meta-analysis was to elucidate the effect of HITHOC on the survival of patients with MPM, as well as to compare the effect size between the various histologic subtypes (epithelioid, biphasic, and sarcomatoid). We also analyzed the rate of reported complications. To our knowledge, this is the first meta-analysis that analyzes the role of HITHOC exclusively to mesothelioma.

## 2. Methods

### 2.1. Design

This is a systematic literature review and meta-analysis that followed a predetermined study protocol according to Preferred Reporting Items for Systematic Reviews and Meta-Analyses (PRISMA) guidelines for systematic reviews and meta-analyses.

### 2.2. Literature Search Strategy

A systematic literature search until January 2021 from multiple databases (Embase, Medline, and Cochrane library) was conducted by the first author (T.J.). The search was performed by combining medical subject headings (MeSH) and related free-text search terms with Boolean operators “AND” or “OR”. The search line used was “Mesotheliom* AND (Hypertherm* AND (intrathorac* OR intrapleur* OR Intracav*) AND Chemother*)”.

### 2.3. Study Selection

The inclusion criteria were as follows: (1) Study including only malignant pleural mesothelioma patients; (2) Study investigating use of hyperthermic intrathoracic/intrapleural chemotherapy; (3) Study reported endpoint of overall survival; (4) Study was done in adults (>18 years of age). Exclusion criteria were: (1) English translation of the manuscript not available.

### 2.4. Data Extraction

Titles and abstracts were scrutinized by the first author (Tommi Järvinen) and duplicates were identified simultaneously. Full texts of potential studies were analyzed by 2 authors (Tommi Järvinen and Ilkka Ilonen). Summary data were extracted from included studies. Extracted data included publication year, sample size, histology, HITHOC agents used, HITHOC temperatures used, length of HITHOC infusion, technique of HITHOC, possible cytoprotective and other adjuncts to HITHOC, operative technique, staging of the tumor, follow-up, full report of complications and length of hospital stay, and overall survival. Authors were contacted directly in order to receive missing data if the study was otherwise eligible for inclusion.

### 2.5. Informed Consent

As a meta-analysis that does not include or process individual patient level data, no informed consent, as per Helsinki University Institutional Review Board guidelines, was needed.

### 2.6. Outcome Measures and Statistical Analysis

The effect size on overall survival was measured using Hedge’s *g*, which was calculated using median overall survival, number of cases in each group, and *p* value.

Meta-analysis of data was conducted using a random effects model due to high heterogeneity. Publication bias was assessed by funnel plots (plots of effect estimates against sample size) to detect outliers or asymmetry. Funnel plot asymmetry was analyzed visually and by Egger test for small-study effects and publication bias. The statistical significance for Egger test was set at *p* < 0.10, as originally described by Egger et al. [17]. Forest plots, i.e., graphical display of estimated odds ratios and 95% confidence intervals, and summary statistics were used to elucidate the results of the studies.

The I2 test was used to evaluate statistical heterogeneity, also known as the outcome variability, in excess of what would be expected due to measurement error alone of the included studies, with levels of heterogeneity defined as not important (I^2^ = 0–25%), moderate (I^2^ = 25–50%), substantial (I^2^ = 50–75%), or considerable (I^2^ = 75–100%).

Significance level used was *p* < 0.05. Statistical analysis was performed with R (R Core Team (2020). R: A language and environment for statistical computing. R Foundation for Statistical Computing, Vienna, Austria. URL https://www.R-project.org/ acessed on 1 January 2020)

## 3. Results

**Figure 1:**
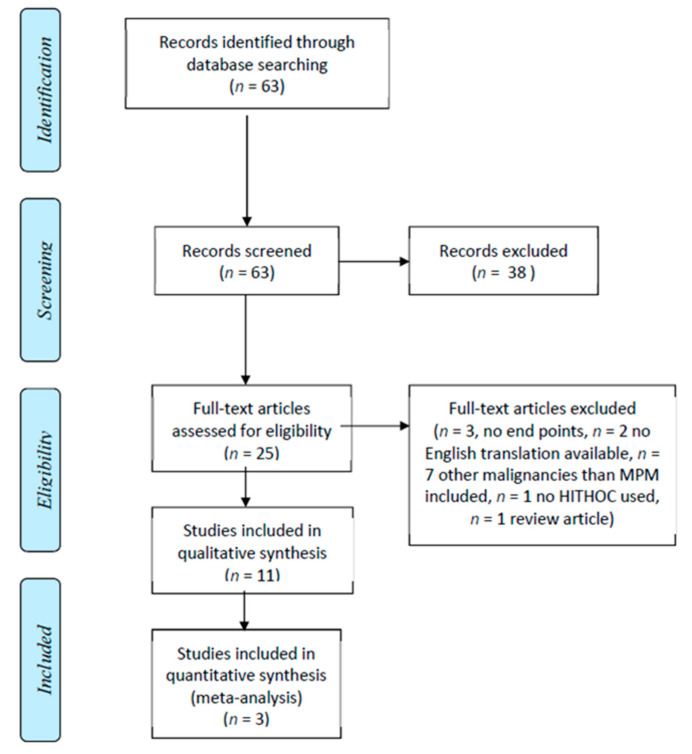
Flow chart of database search and literature selection, as per Preferred Reporting Items for Systematic Reviews and Meta-Analyses (PRISMA) guidelines.

A total of 63 original manuscripts were identified by our search strategy. Figure 1 outlines the inclusion process, as per Preferred Reporting Items for Systematic Reviews and Meta-Analyses (PRISMA) guidelines.

A review of abstracts and titles yielded 24 manuscripts for further evaluation, of which 11 were included in the systematic review. A total of 13 studies were excluded after full text review, as seven studies included other malignancies than MPM (such as thymoma, lung cancer, and mesenchymal malignancies), three studies did not report the prespecified end points, two studies had no English translations available, one study did not use HITHOC in any of the patients, and, lastly, one study for being a review article. Six studies originated from the USA [7,10,18,19,20,21], three from Italy [12,13,22], one from the Netherlands [23], and one from Germany [24]. All of the included studies were observational in nature, and no randomized data regarding this subject are published. These studies and the number of treated patients, their surgical approaches, and group comparisons that could be extracted from the articles are outlined in Table 1. The details of HITHOC treatments are shown in Table 2.

**Figure 2:**
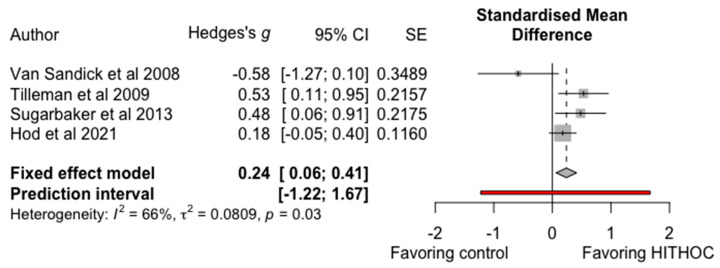
Forest plot of HITHOC vs. no HITOC.

HITHOC was compared to control arm that did not receive HITHOC in four studies, including 762 patients [7,19,20,23]. Van Sandick et al. [23] compared EPP or P/D with HITHOC to EPP with hemithoracic radiation, their intrapleural chemotherapy regimen was cisplatin (fixed dose of 80 mg/m^2^) and adriamycin (dose starting at 20 mg/m^2^, with increments of 5 mg/m^2^ per dose step) at 40–41 °C for 90 min. Tilleman et al. [7] performed EPP and used a 1 h lavage of the chest and abdomen with cisplatin (225 mg/m^2^) at 42 °C, with intravenous sodium thiosulfate, with or without amifostine as a cytoprotective agent. Sugarbaker et al. [20] compared patients who had undergone either EPP or P/D and been given neoadjuvant/adjuvant treatment (control group) or HITHOC (cisplatin 175 to 225 mg/m^2^ for a 1 h lavage at 42 °C, with sodium thiosulfate rescue and/or amifostine protection). This study also included only MPM with epithelioid histology at needle biopsy; however, 16 of the patients were found to have biphasic histology in the final pathology report [20]. Hod et al. [19] compared HITHOC to no HITHOC in regards to AKI. The authors were contacted in order to receive the data needed for inclusion in the meta-analysis. Their HITHOC regimen used cisplatin (175–225 mg/m^2^) and gemcitabine (900 mg/m^2^) with amifostine and sodium thiosulfate protection. The pooled analysis of these studies revealed a standardized mean difference (SMD) of 0.24, with 95% CI of 0.06–0.41 favoring the HITHOC group. Figure 2 shows the associated forest plot. There was marked study heterogeneity I^2^ = 66%, *p* = 0.03.

**Figure 3:**
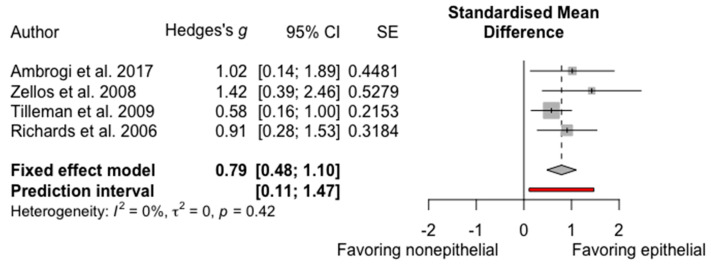
Forest plot of epithelial histology vs. nonepithelial histology.

The survival effect of HITHOC in epithelioid MPM vs. non-epithelioid MPM was analyzed in four studies [7,10,12,18]. Richards et al. [18] studied 24 patients with epithelioid type and 20 with non-epithelioid. Ambrogi et al. [12] reported 43 epithelioid and 6 non-epithelioid tumors. Zellos et al. [10] included 24 epithelioid types and 5 non-epithelioid types. Tilleman et al. [7] had 53 patients with epithelioid, 36 with biphasic, and 5 with sarcomatoid histology. All studies reported an improved survival in epithelioid MPM compared to non-epithelioid MPM. Pooled analysis showed an SMD of 0.79 (95% CI = 0.48–1.10), favoring epithelioid MPM, as shown in Figure 3. As per Cohen et al. [25], an SMD of 0.79 can be interpreted as a borderline “large” effect. Heterogeneity was negligible I^2^ = 0%, *p* = 0.42.

### Complications Related to HITHOC

Of the 11 studies included in this systematic review and meta-analysis, only 1 study did not report any data related to complications [20]. However, the reporting and definition of complications is very heterogeneous between the studies, and two studies reported only the total amount of complications and one study only the incidence of AKI [13,19]. Reported rates of some major complications related to the treatments given are shown in Table 3. The rate of complications varies between 16.7 and 62.9%. Among the most common complications are cardiac complications (7.7–34.1%), such as AF, myocardial infarction, pulmonary embolism, pericarditis, and cardiac dysfunction. Acute kidney injury, mostly related to HITHOC, varied greatly between studies, with the highest incidence with Zellos et al. [10] (75.7%) and lowest with Tilleman et al. [7] (3.3%). Reoperation rates also had much variance, as the lowest rate reported was 2.0% with Ambrogi et al. [12] and the highest incidence was 40% with van Sandick et al. [23]. Median hospital stay varied between 8 and 26 days.

## 4. Discussion

A statistically significant signal of prolonged survival with HITHOC was reached in this meta-analysis. Epithelioid histologic subtype seems to have a better prognosis when treated with HITHOC.

Currently, surgery, namely EPP or P/D, is the main treatment for limited disease MPM. Some studies in this review used partial pleurectomy (pericardium, lung, and diaphragm sparing) as the surgical treatment of choice [12,13]. Other therapies, such as radiotherapy, chemotherapy, and chemoradiation, have been combined with surgery as preoperative, perioperative or postoperative adjuncts to enhance the therapeutic effect of surgery, as well as to control the high recurrence rate of MPM after only surgery.

Several peri- or intraoperative intracavitary therapies have been proposed in order to improve loco-regional effect of surgery. The rationale for intrathoracic therapies is to spread cytotoxic agents on the microscopic tumor surface, where a direct and more efficient effect can be achieved, limiting the systemic adverse effects [13]. In addition to HITHOC, antiseptic povidone [26], photodynamic therapy [27], and immunotherapies [28] have been used. However, even if some of the preliminary findings are encouraging, results are still based largely on low-quality retrospective data, and intracavitary treatments are recommended to be used within a clinical trial [29].

The most popular cytotoxic drugs used for HITHOC were cisplatin followed by doxorubicin and mitomycin C, and 41–43 °C was most commonly used in HITHOC. The standard time for infusion was 60–90 min across the studies. Intrathoracic instillation of chemotherapeutic agents allows for a much higher concentration of the drug in the pleural cavity when compared to systemic chemotherapy, potentially improving the cytotoxic effect to the tumor cells and minimizing systemic adverse effects. The potential benefits of intracavitary chemotherapeutic infusion are numerous, and include increased penetration of chemotherapy into tumor, delayed cavitary clearance of chemotherapy after direct instillation, and increased cytotoxicity with selected chemotherapy agents [30,31]. In theory, the efficacy of chemotherapy is improved by hyperthermia, as local drug absorption increases and chemotherapeutic drug action enhances [30]. The biological mechanism behind the effect of hyperthermia is thought to be protein denaturation of the cancer cells at a temperature of 44 °C for 1 h, while nonmalignant tissues are relatively unharmed at this temperature [31]. This protein denaturation of cancer cells, in turn, results in a rise in the rate of tumor cell apoptosis. This is mainly conveyed through alteration in the DNA synthesis of the cell, cell membrane cytoskeleton, and membrane permeability [14]. Its benefit in the treatment of mucinous appendiceal neoplasms, colorectal cancer peritoneal metastases, gastric cancer carcinomatosis, and ovarian cancer is rather well established [32,33,34,35,36]. However, intrapleural chemoperfusion has a significant risk of comorbidity, especially the risk of acute kidney injury (AKI), and there seems to be a dose-dependent risk [19]. The pharmacokinetics of intrathoracic instillation of mitomycin C and doxorubicin differ from intrapleural instillation, as the intrathoracic absorption efficiency seems to be about half of that of intrapleural absorption, even when comparable and consistently high concentrations are maintained [11]. Unfortunately, no randomized controlled trials on the use of hyperthermic intracavitary chemotherapy exist in pleural malignancies. Thus, this meta-analysis highlights the importance for such, as the results are nonuniform and there is no consensus for this matter.

A previous systematic review by Zhao et al. [37] on this subject found a statistically significant effect favoring HITHOC in the treatment of pleural malignancies. This meta-analysis included five studies in its qualitative synthesis. One of the studies included treated patients with other pleural malignancies than MPM, such as thymoma and lung cancer, and, thus, by our inclusion criteria, this study could not be included in the summary statistics [38]. Zhao et al. [37] also included a study that did not use HITHOC, but hyperthermic pleural lavage with povidone–iodine, and, thus, was excluded from our analysis [26]. As a result, only three studies were included in the analysis. However, the authors of a recent study by Hod et al. [19] were gracious enough to provide us with the data needed in order to include their results in our meta-synthesis [19]. Of the included studies, Tilleman et al. [7], Sugarbaker et al. [20], and Hod et al. [19] reported improved survival with surgery with HITHOC vs. surgery without HITHOC. Van Sandick et al. [23] reported markedly worse survival with HITHOC and either EPP or P/D without radiation therapy (RT) compared to EPP, no HITHOC, and hemithoracic RT. Thus, in this study, the effects of HITHOC on survival are greatly confounded by the difference of other treatment modalities between the study groups [23].

We also tried to analyze the importance of histologic subtype, epithelioid, sarcomatoid, or biphasic being the main subtypes, of MPM for HITHOC therapy. Epithelioid subtype of MPM has been described previously to have a much better overall prognosis than sarcomatoid or biphasic MPM [39]. Although a statistically significant benefit of HITHOC in the treatment of all patients could not be established in this meta-analysis, the effect on only epithelioid subtype remains unknown, as none of the included studies that compared HITHOC to no HITHOC reported survival statistics of only epitheloid subtype [7,20,23]. However, our analysis confirms previous findings that histologic subtype affects survival on surgically treated patients, with epithelioid subtype having clearly better OS than sarcomatoid and biphasic subtypes [40].

The rate of complications following surgery and HITHOC seem to differ a great deal between the studies, as shown in Table 2. This is most likely a reporting issue, as reported complications were not uniform between the studies, and some studies reported no complications or only total amount of complications. The definition and diagnosis of complications most likely is heterogeneous between the studies, but for the exception of Hod et al. [19] with AKI, no studies went into detail on definition of their complications. Nevertheless, the overall rate of complications and morbidity seems to be quite high, ranging from 16.7 to 62.9%, and all but one study reporting approximately half of patients suffering from complications, as shown in Table 2. While some of the complications reported, such as bronchopleural fistulae, diaphragm rupture, and laryngeal nerve dysfunction, are likely attributable to the surgical treatment, such as EPP or P/D, given, it is mostly not possible to deduce from the included studies whether the complications are related to the surgical procedure or administration of HITHOC or both. Indeed, complications from HITHOC per se have been postulated to be quite low, as Liu et al. [41] recently reported incidence of side effects of HITHOC to be 2.0% in 1510 treatments on 315 patients with malignant pleural effusion. The HITHOC in this study was performed under local anesthesia and puncture technique, at bedside, with no associated surgical intervention [41]; 30-day mortality after bedside HITHOC was zero, and most common complications were related to the pleural puncture (pneumothorax, pain at puncture site) [41]. Judging by these findings, HITHOC itself is relatively safe and well tolerated, and, in this study by Liu et al. [41], this method of bedside HITHOC was superior to conventional intraoperative HITHOC.

There are numerous limitations to this meta-analysis. No RCTs comparing HITHOC and surgery to other multimodality therapies, or even surgery alone, exist, so the meta-analysis synthesizes only retrospective data. For this reason, it is subject to the same biases as the studies included and has a significant risk of amplifying them. Moreover, only three of the eleven studies could be enrolled in the qualitative synthesis of overall survival between HITHOC vs. no HITHOC, with a limited number of patients, and only four of the studies could be included in the qualitative synthesis of overall survival between histologic subtypes. The studies included in this meta-analysis suffer from heterogeneity, as the studies and even the groups within the studies have differences in surgeries used, other treatment modalities (such as RT) used, chemotherapeutic agents used in HITHOC, HITHOC temperatures, and HITHOC durations, as shown in Table 1 [7,20,23]. However, due to low volume of the cases and declining incidence in the future, there are no prospective trials to address these study questions on the horizon.

Based on available data, we were not able draw any definitive conclusion of the benefit of HTHOC in treatment of all mesothelioma patients, although there seems to be a statistically significant effect in favor of HITHOC. It is obvious that patients with epithelioid subtype have a better prognosis after HITHOC treatment than with other subtypes, but whether this difference is of significance in overall prognosis remains unclear because of the poor quality of accessible data. Multicenter randomized controlled trials are needed to validate and standardize this treatment approach.

## 5. Conclusions

There is a signal of benefit with HITHOC in MPM patients, but higher quality data are needed to make a definitive conclusion.

## Figures and Tables

**Table 1:** Studies included in the systematic review.

Author	Year	Country	*N*	Treatment	Groups Compared
Richards et al. [18]	2006	USA	44	P/D * and HITHOC ^†^	Histologic subtypes
van Sandick et al. [23]	2008	Netherlands	35	EPP ^‡^ or P/D ^*^ with HITHOC ^†^ or EPP with hemithoracic RT ^§^	HITHOC ^†^ vs. no-HITHOC ^†^
Zellos et al. [10]	2009	USA	29	EPP ^‡^ with HITHOC ^†^	Histologic subtypes, tumor stage
Tilleman et al. [7]	2009	USA	121	EPP ^‡^ with HITHOC ^†^	HITHOC ^†^ vs. no HITHOC ^†^, histologic subtypes, tumor stage
Sugarbaker et al. [20]	2013	USA	103	EPP ^‡^ or P/D * with HITHOC ^†^ with or without HITHOC ^†^	HITHOC ^†^ vs. no-HITHOC ^†^
Migliore et al. [22]	2015	Italy	6	P/D * and HITHOC ^†^	N/a
Bertoglio et al. [13]	2017	Italy	26	Partial pleurectomy	Tumor stage
Burt et al. [21]	2018	USA	104	EPP ^‡^ or P/D * with HITHOC ^†^	EPP ^‡^ vs P/D *
Ambrogi et al. [12]	2018	Italy	49	Partial pleurectomy	Histologic subtypes
Klotz et al. [24]	2019	Germany	71	P/D * with HITHOC ^†^	Histologic subtypes, resection completeness
Hod et al. [19]	2021	USA	503	EPP ^‡^ or P/D * with or without HITHOC ^†^	Acute kidney injury stages

* Pleurectomy and/or decortication; ^†^ hyperthermic intrapleural chemotherapy; ^‡^ extrapleural pneumonectomy; ^§^ radiotherapy.

**Table 2:** Details of hyperthermic intrathoracic chemotherapies used.

Author	Agents	Dose	Temperature	Duration (min)	Adjuncts
Richards et al. [18]	cisplatin	Escalation from 50 mg/m^2^ to 250 mg/m^2^	42 °C	60	Intravenous sodium thiosulfate
van Sandick et al. [23]	cisplatin, adriamycin	80 mg/m^2^, 20 mg/m^2^	40–41 °C	90	
Zellos et al. [10]	cisplatin	910 mg/m^2^	42 °C	60	Intravenous amifostine
Tilleman et al. [7]	cisplatin	225 mg/m^2^	42 °C	60	Intravenous sodium thiosulfate and amifostine
Sugarbaker et al. [20]	cisplatin	175–225 mg/m^2^	42 °C	60	Intravenous sodium thiosulfate and amifostine
Migliore et al. [22]	cisplatin	120 mg/m^2^	42.5 °C	60	
Bertoglio et al. [13]	cisplatin, doxorubicin	80 mg/m^2^, 25 mg/m^2^	42.5 °C	60	
Burt et al. [21]	cisplatin	175–225 mg/m^2^	42 °C	60	Intravenous sodium thiosulfate and amifostine
Ambrogi et al. [12]	cisplatin, epirubicin	80 mg/m^2^, 25 mg/m^2^	42.5 °C	60	
Klotz et al. [24]	cisplatin, doxorubicin	200 mg, 100 mg	42 °C	90	
Hod et al. [19]	cisplatin, gemcitabine	175–225 mg/m^2^, 900 mg/m^2^	42 °C	60	Intravenous sodium thiosulfate and amifostine

**Table 3:** Complications reported in studies.

Author	Any Complication (%)	Pneumonia (%)	Pneumo- thorax (%)	Empyema (%)	ARDS * (%)	AKI ^●^ (%)	Cardiac Complication ^Ω^ (%)	Reoperation (%)	Median Hospital Stay (Days)
Richards et al. [18]	N/A	9.1	N/a	N/A	11.4	43.9	34.1	4.5	11
van Sandick et al. [23]	62.9	N/A	N/a	8.6	N/A	N/A	31.4	40.0	15
Zellos et al. [10]	N/A	10.3	N/a	10.3	28.6	75.7	N/A	20.7	15
Tilleman et al. [7]	48.9	3.3	N/a	2.2	6.5	3.3	29.3	N/A	12
Sugarbaker et al. [20]	N/A	N/A	N/a	N/A	N/A	N/A	N/A	N/A	12
Migliore et al. [22]	16.7	N/A	N/a	N/A	N/A	N/A	N/A	N/A	N/A
Bertoglio et al. [13]	50.0	N/A	N/a	N/A	N/A	N/A	N/A	N/A	N/A
Burt et al. [21]	57.7	N/A	1.9	1.0	3.8	4.8	7.7	N/A	N/A
Ambrogi et al. [12]	46.9	N/A	N/a	2.0	N/A	N/A	N/A	2.0	8
Klotz et al. [24]	57.7	16.9	28.2	N/A	N/A	N/A	23.9	14.1	26
Hod et al. [19]	N/A	N/A	N/A	N/A	N/A	48.3	N/A	N/A	N/A

* Adult respiratory distress syndrome; ^●^ acute kidney injury; ^Ω^ atrial fibrillation, heart failure, myocardial infarction, pericarditis, pulmonary embolism; N/A-not available.

## Data Availability

The R code used for data analysis can be requested by contacting the corresponding author; no other original data was involved in this study.
